# Frequency of Renal Monitoring — Creatinine and Cystatin C (FORM-2C): an observational cohort study of patients with reduced eGFR in primary care

**DOI:** 10.3399/BJGP.2020.0940

**Published:** 2021-07-13

**Authors:** Susannah Fleming, Rafael Perera-Salazar, Kathryn S Taylor, Louise Jones, FD Richard Hobbs, Tim James, Chris A O’Callaghan, Brian Shine, Jan Y Verbakel, Richard Stevens, Clare Bankhead

**Affiliations:** Nuffield Department of Primary Care Health Sciences, University of Oxford, Oxford, UK.; Nuffield Department of Primary Care Health Sciences, University of Oxford, Oxford, UK; National Institute for Health Research (NIHR) Oxford Biomedical Research Centre, Oxford, UK; professor of medical statistics, NIHR Oxford and Thames Valley Applied Research Collaborative, Oxford, UK.; Nuffield Department of Primary Care Health Sciences, University of Oxford, Oxford, UK.; Nuffield Department of Primary Care Health Sciences, University of Oxford, Oxford, UK.; Nuffield Department of Primary Care Health Sciences, University of Oxford, Oxford, UK; Nuffield professor of primary care health sciences, National Institute for Health Research (NIHR) Oxford Biomedical Research Centre, Oxford, UK; NIHR Oxford and Thames Valley Applied Research Collaborative, Oxford, UK.; Department of Clinical Biochemistry, Oxford University Hospitals NHS Foundation Trust, Oxford, UK.; NIHR Oxford Biomedical Research Centre, Oxford, UK; Nuffield Department of Medicine, University of Oxford, Oxford, UK.; Department of Clinical Biochemistry, Oxford University Hospitals NHS Foundation Trust, Oxford, UK.; Nuffield Department of Primary Care Health Sciences, University of Oxford, Oxford, UK; assistant professor, EPI-Centre, Department of Public Health and Primary Care, KU Leuven, Belgium.; Nuffield Department of Primary Care Health Sciences, University of Oxford, Oxford, UK.; Nuffield Department of Primary Care Health Sciences, University of Oxford, Oxford, UK; National Institute for Health Research (NIHR) Oxford Biomedical Research Centre, Oxford, UK; NIHR Oxford and Thames Valley Applied Research Collaborative, Oxford, UK.

**Keywords:** chronic kidney diseases, creatinine, cystatin C, estimated glomerular filtration rate, primary care

## Abstract

**Background:**

Monitoring is the mainstay of chronic kidney disease management in primary care; however, there is little evidence about the best way to do this.

**Aim:**

To compare the effectiveness of estimated glomerular filtration rate (eGFR) derived from serum creatinine and serum cystatin C to predict renal function decline among those with a recent eGFR of 30–89 ml/min/1.73 m^2^.

**Design and setting:**

Observational cohort study in UK primary care.

**Method:**

Serum creatinine and serum cystatin C were both measured at seven study visits over 2 years in 750 patients aged ≥18 years with an eGFR of 30–89 ml/min/1.73 m^2^ within the previous year. The primary outcome was change in eGFR derived from serum creatinine or serum cystatin C between 6 and 24 months.

**Results:**

Average change in eGFR was 0.51 ml/min/1.73 m^2^/year when estimated by serum creatinine and −2.35 ml/min/1.73 m^2^/year when estimated by serum cystatin C. The c-statistic for predicting renal decline using serum creatininederived eGFR was 0.495 (95% confidence interval [CI] = 0.471 to 0.519). The equivalent c-statistic using serum cystatin C-derived eGFR was 0.497 (95% CI = 0.468 to 0.525). Similar results were obtained when restricting analyses to those aged ≥75 or <75 years, or with eGFR ≥60 ml/min/1.73 m^2^. In those with eGFR <60 ml/min/1.73 m^2^, serum cystatin C-derived eGFR was more predictive than serum creatinine-derived eGFR for future decline in kidney function.

**Conclusion:**

In the primary analysis neither eGFR estimated from serum creatinine nor from serum cystatin C predicted future change in kidney function, partly due to small changes during 2 years. In some secondary analyses there was a suggestion that serum cystatin C was a more useful biomarker to estimate eGFR, especially in those with a baseline eGFR <60 ml/min/1.73 m^2^.

## INTRODUCTION

Given the benefits of health promotion, advancements in medical care, and improved disease prevention, most people will eventually live with a chronic condition, such as kidney disease. Chronic kidney disease (CKD) is associated with increased cardiovascular risk (predominantly stroke, ischaemic heart disease, and heart failure), and increased all-cause mortality.^1^^–^^5^

Prevalence rates from Quality and Outcomes Framework returns indicate that 4.1% of adults in England are recorded as having CKD stages 3–5.^6^ However, this is likely to be an underestimate, as recent modelling of CKD stages 3–5 estimates that 2.6 million people (95% confidence interval [CI] = 2.3 to 3.0) aged ≥16 years are living with diagnosed or undiagnosed CKD stages 3–5 in England, representing 6.1% (95% CI = 5.3% to 7.0%) of the population in this age group.^7^ This is more consistent with the median worldwide prevalence of 7.2% (range 1.5%–43.3%) from studies of CKD stages 3–5.^8^

Mildly reduced renal function, although not necessarily diagnosed as CKD, is likely to be more prevalent, as suggested by estimates from US data that approximately 10% of the population have an estimated glomerular filtration rate (eGFR) between 60 and 90 ml/min/1.73 m^2^^;^^9^ a normal eGFR is >90 ml/min/1.73 m^2^.^10^ All these calculations depend to some extent on the estimation method used to calculate eGFR. Before 2014 in the UK, eGFR was estimated using creatinine and the Modification of Diet in Renal Disease (MDRD) equation, but the National Institute for Health and Care Excellence (NICE) has since recommended that the CKD Epidemiology Collaboration (CKD-EPI) equation should be used instead.^10^ NICE has also recommended that eGFR could be estimated using cystatin C, an alternative biomarker, in people with an eGFR-creatinine of 45–59 ml/min/1.73 m^2^ and no other evidence of kidney disease. Although cystatin C can be more expensive to measure, there is evidence that eGFR measures incorporating cystatin C have improved accuracy for predicting outcomes such as death and end-stage renal disease.^11^

**Table:** How this fits in

Mild chronic kidney disease is a common condition in primary care, but it is not clear what the optimal monitoring strategy should be. This research investigated whether serum creatinine or serum cystatin C is able to predict future decline in renal function in patients with mild chronic kidney disease. Although there is an indication that serum cystatin C-derived eGFR is more predictive of future renal decline in patients with eGFR <60 ml/min/1.73 m^2^, there is insufficient evidence to suggest a change in practice. Clinicians should continue to choose an appropriate testing strategy for estimating current GFR, rather than considering the ability to predict future renal decline.

The focus for managing chronic conditions has gradually changed from secondary care to primary care as conditions are recognised earlier in their disease process. Once a chronic condition has been diagnosed, a major aspect of management is monitoring. Notwithstanding economic costs, for many chronic disorders there is no or poor evidence on how, what, and when to monitor.

Most people with CKD (98%) are managed in primary care using a multifactorial approach of repeated monitoring, maintenance of blood pressure below agreed guideline limits (<140/90 mmHg, or <130/80 mmHg for those with diabetes or proteinuria), treatment of hypertension with angiotensin-converting enzyme inhibitors or angiotensin receptor blockers in the presence of proteinuria, and encouragement to lead a healthy lifestyle.^12^

The 2014 NICE guidance recommends that the frequency of monitoring should depend on the patient’s clinical situation, but suggests that monitoring should be annual for those with CKD stages 1 and 2, every 6 months for those with stage 3 disease, every 3 months for stage 4 disease, and 6-weekly for stage 5 disease.^10^

However, these recommended intervals for monitoring CKD are derived from consensus rather than evidence. There is evidence to suggest that for several conditions current monitoring strategies are likely to overestimate changes in disease intensity when they are due only to biological and day-to-day variability.^13^^–^^15^ Although estimates of eGFR ‘noise’ have not been determined, it is likely that the frequency of the monitoring regimen for CKD is suboptimal.

Therefore, more frequent monitoring may not necessarily be better. Besides the financial costs, the considerable downsides to over-frequent measurement for the patient include anxieties about the time spent on testing, having invasive tests, and unnecessary hospital visits. Perhaps more important is the potential for inadequate management based on incorrect test results, showing deterioration when it is absent and vice versa.

Additionally, it is not known whether monitoring CKD using serum creatinine or serum cystatin C is preferable in primary care populations. The 2008 NICE guidelines on the early identification and management of CKD (which have since been superseded) recommended research into identifying *‘more accurate and cost effective methods of monitoring kidney function, especially in patients with GFR 60 ml/min/1.73 m^2^ or more’*.^12^

Therefore, this study compared the effectiveness of serum creatinine or serum cystatin C measures in estimating eGFR at different time intervals, to predict renal function decline (including proteinuria), among those with a recent eGFR of 30–89 ml/min/1.73 m^2^ (CKD stages 2 and 3).

## METHOD

### Study design and participants

A cohort study was used to compare the usefulness of two biomarkers (serum cystatin C and serum creatinine) to calculate eGFR to predict future change in kidney function.

Participants were recruited from 14 general practices in Oxfordshire, Berkshire, and Buckinghamshire in the UK between 12 August 2014 and 15 August 2016. Using the eligibility criteria (see Supplementary Box S1 for details), practices identified potential participants from their electronic health records and CKD registers using a search to identify patients aged ≥18 years whose most recent eGFR blood test, taken within the last 12 months, was between 30 and 89 ml/min/1.73 m^2^. These patients were then contacted by post.

Participants attended an initial visit and a further six scheduled follow-up visits over 2 years at their GP surgery (see Supplementary Figure S1 for details). The final follow-up visit was on 29 November 2018. At each visit, blood samples were taken for measurement of serum creatinine and serum cystatin C.^16^ Early-morning urine samples were analysed after each visit to measure the albumin:creatinine ratio (ACR). Laboratory measures were undertaken using an Abbott c16000 chemistry analyser; serum creatinine by an enzymatic method; and serum cystatin C and urine albumin using immunoturbidimetric assay. Method performance was stable and precise throughout the study period, as evidenced through external quality assurance scheme participation.

Demographic and medical information was collected for all participants, including age, sex, ethnicity, diabetes status, presence of chronic heart failure, blood pressure, height, weight, and waist and hip measurements. Current medications and recent changes in medication were logged at each visit.

### Outcomes

The primary outcome was change in calculated eGFR using the CKD-EPI equations for either serum creatinine or serum cystatin C measurements.^16^ Baseline values of these biomarkers during the first 3 months were averaged, to minimise biological and assay noise in the estimates of initial renal status. To avoid spurious correlation with the initial measures, subsequent values, between 6 months and 2 years, were used to estimate the primary outcome.

Secondary outcomes included proteinuria; rapid progression of CKD, defined as a yearly decline of >5 ml/min/1.73 m^2^ (planned analysis); and final eGFR at 24 months (post-hoc analysis).

### Statistical analysis

Separate analyses were conducted for each of serum creatinine-derived eGFR and serum cystatin C-derived eGFR, for example, serum creatinine-based measures at baseline were used to predict serum creatinine-based outcomes at follow-up.

The predictions of change in the eGFR measure were calculated by carrying out a linear regression within each participant using all non-missing serum creatinineor serum cystatin C-derived eGFR values between 6 months and 2 years for that participant. A c-statistic was calculated to measure the concordance between baseline eGFR and change in eGFR for both serum creatinine and serum cystatin C. Results for all measures were reported with 95% confidence intervals (CIs) calculated by bias-corrected and accelerated bootstrap methods.^17^

All participants with at least one data point for each of serum creatinine and serum cystatin C measured from visits 1–3, and at least two data points for each measure from visits 4–7, were eligible for the primary analysis, which was unstratified. Subgroup analyses were stratified by eligibility eGFR (60–90 ml/min/1.73 m^2^ and 30–60 ml/min/1.73 m^2^) and by age (≥75 years versus <75 years). The eGFR band, corresponding to values used, was used to define CKD stage (see Supplementary Table S1 for details), to determine stratification, and to assess discordant classification between serum cystatin C- and serum creatinine-based eGFR measures, using the categories stated in the NICE 2014 guidance.^10^

Since the number of patients with both serum creatinine and serum cystatin C measurements at 24 months was relatively low, a post-hoc sensitivity analysis was also carried out using the last observation carried forward, beginning at the 6-month visit. As separate post-hoc secondary analyses, the primary analysis was repeated but predicting final eGFR and using the Spearman’s rank correlation coefficient instead of the c-statistic.

Secondary analysis of the rapid progression of CKD was carried out using the pROC package (version 1.16.2) in R, which uses a bootstrap method to calculate 95% CI. The difference between the two c-statistics was calculated as for the primary analysis.

Further details about methods are given in Supplementary Appendix S1. All analyses were carried out using the R statistical computing package (version 3.6.3).

## RESULTS

### Descriptive statistics of population

**Figure 1.**
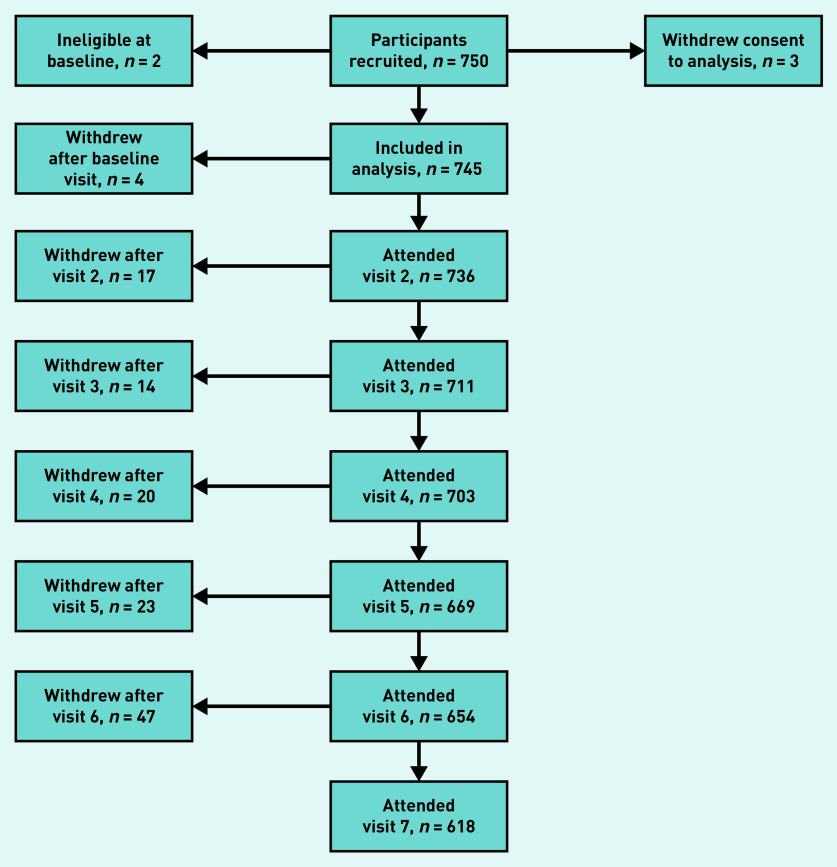
*Participant flow through the FORM-2C study. FORM-2C = Frequency of Renal Monitoring — Creatinine and Cystatin C.*

In total, 750 participants were recruited to the study, but only 745 were available for inclusion in the analysis dataset, because two were found to be ineligible, and three later withdrew consent for analysis. Figure 1 shows the participant flow through the study. The median follow-up time was 2.0 years, with a maximum of 2.6 years, and the total study follow-up time was 1362 person–years.

In total, 128 participants were discontinued from the study, including the three who withdrew consent for analysis. The most commonly cited reason for discontinuation was loss to follow-up (*n* = 71, 55%), followed by changes in health (*n* = 14, 11%), death (*n* = 11, 9%), and moving out of the study area (*n* = 9, 7%). Cause of death was available for six of the 11 participants who died during the study period, and was noted as sepsis in three cases, neoplasm in two cases (one brain tumour, one ovarian neoplasm), and ischaemic heart disease in one case.

As shown in the baseline characteristics of the Frequency of Renal Monitoring — Creatinine and Cystatin C (FORM-2C) population (Table 1), the most common comorbidity in the cohort was hypertension, followed by formal diagnosis of CKD. Note that, as the inclusion criteria were based on a single measurement of eGFR, many participants did not have a formal diagnosis of CKD at baseline. Levels of proteinuria (defined as ACR ≥30 mg/mmol) were very low at baseline, with only five participants (0.7%) demonstrating this in their visit 1 urine samples (data not shown).

**Table 1.** Baseline characteristics of the FORM-2C population (*n* = 745)

**Variable**		**Summary, *n* (%)^a^**	**Missing, *n* (%)^a^**
**Age, years, mean (SD)**		69.8 (9.6)	0 (0.0)

**Sex**	Female	394 (52.9)	3 (0.4)
	Male	348 (46.7)	

**Ethnicity**	White	719 (96.5)	0 (0.0)
	Mixed ethnicity	6 (0.8)	
	Asian	10 (1.3)	
	Black	5 (0.7)	
	Other	5 (0.7)	

**Comorbidities in GP record^b^**	Hypertension	429 (57.6)	n/a
	Chronic kidney disease	204 (27.4)	
	Diabetes	164 (22.0)	
	History of urinary tract infection	122 (16.4)	
	Cancer	112 (15.0)	
	Thyroid disease	89 (11.9)	
	Prostate disorders	85 (11.4)	
	Ischaemic heart disease	82 (11.0)	
	Atrial fibrillation	54 (7.2)	
	Myocardial infarction	44 (5.9)	
	Kidney stones	40 (5.4)	
	Transient ischaemic attack	28 (3.8)	
	Stroke	19 (2.6)	
	Heart failure	15 (2.0)	
	Peripheral vascular disease	14 (1.9)	

**Smoking status**	Never smoked	386 (51.8)	2 (0.3)
	Former smoker	327 (43.9)	
	Current smoker	30 (4.0)	

**Biochemical markers at visit 1, mean (SD)**	Serum creatinine (µmol/l)	88.4 (23.2)	16 (2.1)
	Serum cystatin C (mg/l)	1.2 (0.4)	83 (11.1)
	ACR (mg/mmol)	3.4 (9.4)^c^	51 (6.8)
	eGFR-serum creatinine (ml/min/1.73 m^2^)	69.4 (16.2)	19 (2.6)
	eGFR-serum cystatin C (ml/min/1.73 m^2^)	61.9 (20.3)	86 (11.5)

**eGFR over the extended baseline, mean (SD)**	eGFR-serum creatinine (ml/min/1.73 m^2^)	68.9 (15.5)	6 (0.8)
	eGFR-serum cystatin C (ml/min/1.73 m^2^)	61.1 (19.4)	8 (1.1)

a *Unless otherwise stated.*

b *Those with a diagnosis or history noted in the GP record at the first visit.*

c *ACR summaries do not include measures that were reported by the lab as ‘unable to calculate’ because of very low levels of urine albumin, or those entered as ‘normal’ on the GP system, where it was not possible to obtain a more accurate result. ACR = albumin:creatinine ratio. eGFR = estimated glomerular filtration rate. FORM-2C = Frequency of Renal Monitoring — Creatinine and Cystatin C. n/a = not applicable.*

Table 2 summarises how participants were classified into eGFR ranges using various different definitions of baseline eGFR. The discordance between the classifications of participants is also shown in Supplementary Tables S3 and S4, which show the changes across different periods from 2 weeks.

**Table 2.** Proportions of participants in the FORM-2C cohort (*n* = 745) classified into each eGFR range before the initial visit, at visit 1, and over the extended baseline (mean of values measured at visits 1–3)

**eGFR range (ml/min/1.73 m^2^)**	**Pre-baseline eGFR, *n* (%)**	**eGFR-serum creatinine: visit 1, *n* (%)**	**eGFR-serum creatinine: extended baseline (visits 1–3), *n* (%)**	**eGFR-serum cystatin C: visit 1, *n* (%)**	**eGFR-serum cystatin C: extended baseline (visits 1–3), *n* (%)**
≥90	7 (0.9)	63 (8.5)	47 (6.3)	68 (9.1)	63 (8.5)
≥60 and <90	520 (69.8)	462 (62.0)	487 (65.4)	279 (37.4)	320 (43.0)
≥45 and <60	167 (22.4)	136 (18.3)	144 (19.3)	176 (23.6)	200 (26.8)
≥30 and <45	49 (6.6)	58 (7.8)	57 (7.7)	100 (13.4)	118 (15.8)
<30	0 (0.0)	7 (0.9)	4 (0.5)	36 (4.8)	36 (4.8)
Missing	2 (0.3)	19 (2.6)	6 (0.8)	86 (11.5)	8 (1.1)

*eGFR = estimated glomerular filtration rate. FORM-2C = Frequency of Renal Monitoring — Creatinine and Cystatin C.*

A small number of participants had pre-baseline eGFR measures of >90 ml/min/1.73 m^2^. In these cases, patients had been invited to participate on the basis of an earlier reduced eGFR, but then had another measurement between invitation and baseline visit. The eGFR at the time of invitation was used to determine inclusion, but the most recent eGFR was used as the pre-baseline measure.

### Missing data

Supplementary Table S2 shows the number of participants with missing data for serum creatinine and serum cystatin C at each visit. This may be a result of discontinuation, missed visits, or missing laboratory values. Supplementary Table S2 also gives details of the number of participants with sufficient data to be included in the primary analysis (at least one data point from visits 1–3, and at least two data points from visits 4–7, are required for inclusion).

### Analyses of primary outcome

**Figure 2.**
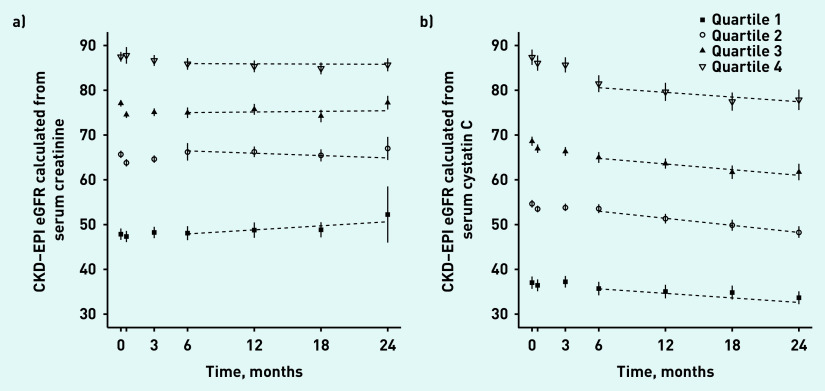
*Mean and 95% CIs for quartiles of a) eGFR-serum creatinine and b) eGFR-serum cystatin C calculated from the FORM-2C cohort at baseline and at 24 months.* *CKD–EPI = Chronic Kidney Disease Epidemiology Collaboration. eGFR = estimated glomerular filtration rate. FORM-2C = Frequency of Renal Monitoring — Creatinine and Cystatin C.*

Using serum creatinine to estimate eGFR, the mean change in eGFR from visits 4 (6 months) to 7 (24 months) was a non-significant (*P* = 0.29) increase of 0.51 ml/min/1.73 m^2^/year (standard deviation [SD] 12.34). Using serum cystatin C to estimate eGFR, the mean change in eGFR for the same period was a statistically significant (*P*<0.001) decrease of 2.35 ml/min/1.73 m^2^ (SD 5.89) (data not shown). Figure 2 illustrates the average change in eGFR by quartile of baseline eGFR.

The c-statistic for baseline eGFR-serum creatinine as a predictor of future change in eGFR-serum creatinine was 0.495 (95% CI = 0.471 to 0.519). The c-statistic for baseline eGFR-serum cystatin C as a predictor of future change in eGFR-serum cystatin C was 0.497 (95% CI = 0.468 to 0.525). The difference in c-statistics (serum creatinine minus serum cystatin C) was −0.00157 (95% CI = −0.0358 to 0.0273). Table 3 shows this analysis stratified by eGFR at recruitment (pre-baseline) and by age.

**Table 3.** Results of primary analysis (*n* = 649) and secondary analyses in the FORM-2C cohort stratified by pre-baseline eGFR (*n* = 641), and separately stratified by age at baseline (*n* = 649)

**Stratum**	***n***	**c-statistic for eGFR-serum creatinine (95% CI)**	**c-statistic for eGFR-serum cystatin C (95% CI)**	**Difference in c-statistics^a^ (95% CI)**
All participants (primary analysis)	649	0.495 (0.471 to 0.519)	0.497 (0.468 to 0.525)	−0.00157 (−0.0358 to 0.0273)
Pre-baseline eGFR ≥60 and <90 ml/min/1.73 m^2^	450	0.492 (0.462 to 0.522)	0.530 (0.500 to 0.563)	−0.0379 (−0.0760 to 0.00289)
Pre-baseline eGFR ≥30 and <60 ml/min/1.73 m^2^	191	0.491 (0.442 to 0.540)	0.412 (0.371 to 0.469)	0.0789 (0.0178 to 0.145)
Aged<75 years	453	0.502 (0.473 to 0.529)	0.516 (0.484 to 0.546)	−0.0135 (−0.0492 to 0.0257)
Aged ≥75 years	196	0.496 (0.452 to 0.543)	0.448 (0.402 to 0.499)	0.0478 (−0.0135 to 0.109)

a *Serum creatinine minus serum cystatin C. eGFR = estimated glomerular filtration rate. FORM-2C = Frequency of Renal Monitoring — Creatinine and Cystatin C.*

The non-significant difference between the c-statistics for serum creatinine and serum cystatin C was also obtained when the analysis was repeated using the post-hoc analysis with Spearman’s rank correlation coefficient (ρ) (see Supplementary Table S5 for details).

### Further analyses

Proteinuria values were stable over time. The results for the ability of baseline eGFR to predict rapid progression (yearly decline of >5 ml/min/1.73 m^2^) of CKD and final eGFR at 24 months are shown in Supplementary Table S6. The c-statistics for rapid progression were 0.475 (95% CI = 0.424 to 0.527) for serum creatinine and 0.628 (95% CI = 0.547 to 0.709) for serum cystatin C.

## DISCUSSION

### Summary

Current eGFR does not predict future change in kidney function over a 2-year period, and neither eGFR based on serum creatinine nor on serum cystatin C provides a better prediction of future change in eGFR. However, in a pre-specified subgroup analysis, eGFR-serum cystatin C was weakly predictive of future change in those with an eGFR <60 ml/min/1.73 m^2^ at recruitment, as evidenced by a 95% CI not crossing the 0.5 value indicating prediction equivalent to chance, although this was not seen in other subgroups. In a secondary analysis looking at rapid progression of CKD, eGFR-serum cystatin C (but not eGFR-serum creatinine) was weakly predictive of future decline in kidney function.

In this relatively short 2-year time period, there was no significant change in kidney function (on average), when measured by eGFR-serum creatinine, whereas a slight decrease could be detected using eGFR- serum cystatin C. This may explain why both measures generally performed poorly at predicting a change in kidney function; however, in a post-hoc analysis, both eGFR-serum creatinine and eGFR-serum cystatin C were shown to be good predictors of kidney function after 2 years.

### Strengths and limitations

This study used a large, well-characterised cohort of patients with reduced eGFR, with good follow-up rates over 2 years despite an average age of 70 years and high levels of comorbidity. Despite missing data, it was possible to include nearly all non- discontinued participants in the primary analysis.

Generalisability of the results to other populations may be limited because of the low ethnic variation in the cohort: this reflects the ethnic mix of the areas in which participants were recruited. In addition, although the inclusion criteria allowed eGFRs of 30–89 ml/min/1.73 m^2^, only a small number of participants with eGFRs <45 ml/min/1.73 m^2^ (CKD stage 3B) were recruited, and so the general results may not be as applicable to this subgroup.

Even where c-statistics in the results for predicting renal decline were significantly different from 0.5 from a statistical point of view, they still represented relatively poor predictive ability.

### Comparison with existing literature

This study has similarities to an ongoing study, the eGFR-C study,^18^ which also aims to assess the value of creatinineand cystatin C-derived eGFR for predicting future renal decline. It is intended that the eGFR-C study will include a higher proportion of non-white participants, as well as participants with baseline proteinuria, who were poorly represented in the current FORM-2C study, and will be followed up over 3 years. The authors therefore hope that the results of the eGFR-C study will extend the generalisability of the results to a wider demographic.

### Implications for research and practice

The 2014 update to the NICE guidelines for assessment and management of CKD^10^ did not recommend superseding creatinine with cystatin C for routine estimation of GFR. Instead, the recommendations stated that interpretation of creatininebased estimations should be interpreted with caution in specific populations such as people with extremes of muscle mass (and conversely to cautiously interpret cystatin C-based estimations in people with uncontrolled thyroid disease).^10^

The results of the current study are consistent with this guidance for clinicians, because serum cystatin C was not superior in the primary analysis, and only weakly superior in some, but not all, secondary analyses. The authors would, therefore, suggest that clinicians should make decisions on which test to use on the basis of the suitability of the test for estimation of current GFR for each particular patient, rather than any general assumption about the ability of any such test to predict future renal decline. Clinicians should also take into account the considerably higher cost of measuring cystatin C compared with creatinine.

Further follow-up of this cohort to 10 years from baseline is planned; this will allow the authors to capture more events such as transitions to later stages of CKD, and associated morbidity and mortality. This may also help confirm the suggestion in some secondary analyses of modest advantages for serum cystatin C over serum creatinine in estimating future renal function decline.

## References

[1] Go AS, Chertow GM, Fan D (2004). Chronic kidney disease and the risks of death, cardiovascular events, and hospitalization. N Engl J Med.

[2] James MT, Hemmelgarn BR, Tonelli M (2010). Early recognition and prevention of chronic kidney disease. Lancet.

[3] O’Hare AM, Bertenthal D, Covinsky KE (2006). Mortality risk stratification in chronic kidney disease: one size for all ages?. J Am Soc Nephrol.

[4] Rifkin DE, Shlipak MG, Katz R (2008). Rapid kidney function decline and mortality risk in older adults. Arch Intern Med.

[5] Wen CP, Cheng TYD, Tsai MK (2008). All-cause mortality attributable to chronic kidney disease: a prospective cohort study based on 462 293 adults in Taiwan. Lancet.

[6] NHS Digital (2018). Quality and Outcomes Framework (QOF), England: 2017–18, prevalence, achievement and exceptions report..

[7] Public Health England (2014). Chronic kidney disease prevalence model.

[8] Zhang QL, Rothenbacher D (2008). Prevalence of chronic kidney disease in population-based studies: systematic review.. BMC Public Health.

[9] Levey AS, Coresh J (2012). Chronic kidney disease. Lancet.

[10] National Institute for Health and Care Excellence (2015). Chronic kidney disease in adults: assessment and management CG182.

[11] Shlipak MG, Matsushita K, Ärnlöv J (2013). Cystatin C versus creatinine in determining risk based on kidney function. N Engl J Med.

[12] National Collaborating Centre for Chronic Conditions (2008). Chronic kidney disease: early identification and management of chronic kidney disease in adults in primary and secondary care CG73.

[13] Buclin T, Telenti A, Perera R (2011). Development and validation of decision rules to guide frequency of monitoring CD4 cell count in HIV-1 infection before starting antiretroviral therapy. PLoS One.

[14] Glasziou PP, Irwig L, Heritier S (2008). Monitoring cholesterol levels: measurement error or true change?. Ann Intern Med.

[15] Oke JL, Stevens RJ, Gaitskell K, Farmer AJ (2012). Establishing an evidence base for frequency of monitoring glycated haemoglobin levels in patients with Type 2 diabetes: projections of effectiveness from a regression model. Diabet Med.

[16] Inker LA, Schmid CH, Tighiouart H (2012). Estimating glomerular filtration rate from serum creatinine and cystatin C. N Engl J Med.

[17] Efron B, Tibshirani RJ (1998). An introduction to the bootstrap.

[18] Lamb EJ, Brettell EA, Cockwell P (2014). The eGFR-C study: accuracy of glomerular filtration rate (GFR) estimation using creatinine and cystatin C and albuminuria for monitoring disease progression in patients with stage 3 chronic kidney disease — prospective longitudinal study in a multiethnic population.. BMC Nephrol.

